# ROX index as a predictor of failure of high-flow nasal cannula in infants with bronchiolitis

**DOI:** 10.1038/s41598-024-51214-4

**Published:** 2024-01-03

**Authors:** Milena Siciliano Nascimento, Bianca Agostini Zólio, Luciana Assis Pires Andrade Vale, Patrícia Angelica de Lima Silva, Thereza Silva Souza, Louise Helena Rodrigues Gonçalves, Linus Pauling Fascina, Cristiane do Prado

**Affiliations:** 1 Departamento de Práticas Assistenciais, Hospital Israelita Albert Einstein, Avenue Albert Einstein, 627-701, São Paulo, SP 05651-901 Brazil; 2 Departamento Materno-Infantil, Vila Santa Catarina Municipal Hospital, São Paulo, Brazil; 3 Departamento Materno-Infantil, Hospital Israelita Albert Einstein, São Paulo, Brazil

**Keywords:** Respiratory tract diseases, Paediatric research

## Abstract

High-flow nasal cannula (HFNC) is a relatively recent therapy that has been used to treat respiratory failure. Until now, the criterion for failure requiring escalation to other forms of ventilatory support has remained unclear. This study evaluated how the ROX index predicts the success or failure of HFNC in infants with bronchiolitis. A prospective, observational, multicenter study was conducted in 2 pediatric ICUs. The data were collected at 7 moments. Patients were categorized into failure and success groups according to HFNC. A total of 102 infants were included, 18(17.6%) of whom failed HFNC therapy. For the ROX index, significant differences were observed between the failure 5.8(95%CI 4.7–7.1) and success 7.7(95%CI 7.2–8.2) groups (*p* = 0.005) at the 12 h evaluation. According to the analysis of the performance of the ROX index, the AUC at 12 h was 0.716(95%CI 0.591–0.842; *p* = 0.016). The best cutoff range for the ROX index at 12 h was 6.50–7.18, with a sensitivity of 42% and a specificity of 66% at the cutoff of 6.50, and a sensitivity of 92% and a specificity of 54% at the cutoff of 7.18. We concluded that the ROX index could be effective at predicting the failure of HFNC therapy in infants with bronchiolitis beginning at 12 h after installation.

## Introduction

High-flow nasal cannula (HFNC) therapy is a relatively recent therapy that has been used to treat respiratory failure^1–3^. Its use has been associated with improved oxygenation, decreased effort and a decreased respiratory rate^4^ and is reflected in the need for escalation of respiratory support, such as the use of noninvasive ventilation (NIV) and orotracheal intubation^5,6^. While the use of HFNC does not substitute for NIV, its indication in the early stages of respiratory failure can effectively prevent clinical deterioration. Several studies that looked for predictors based on clinical signs did not obtain consistent results because what was significant in one study was not confirmed by others^5,7–9^. Although the evaluation of clinical signs such as SpO_2_, FiO_2_, SpO_2_/FiO_2_, respiratory rate, and respiratory distress has guided decision-making and several authors have sought predictors to determine therapy failure, the criteria for discontinuation have not been fully defined; moreover, to date, the literature does not provide a gold standard for predicting HFNC failure^7–9^. Given this gap in knowledge, the aim of our study was to evaluate a new index called the ROX as a predictor of the success or failure of HFNC therapy in infants with bronchiolitis, specifically, whether the ROX index is able to determine the need for a change in respiratory support.

In 2016, Roca et al.^10^ introduced the ROX index, a novel measure utilizing respiratory variables. The index aims to identify patients unresponsive to HFNC. In the numerator, variables with a positive association with HFNC success, such as oxygenation, were placed and assessed by the SpO_2_/FIO_2_ ratio. In contrast, the RR was placed in the denominator because it has an inverse association with HFNC success. The authors used the name respiratory rate -oxygenation (ROX) for the index as the ratio between the S/F ratio and the RR. The ROX index (SpO_2_/FiO_2_)/RR showed good accuracy as a predictor of the need for IMV in adult patients, with a cutoff value of 4.88 at 12 h . Since then, other authors have studied the aim of helping decision-making at the bedside in a practical and reliable way, including during the COVID-19 pandemic^11–15^.

In the pediatric population, Yildizdas et al. defined pediatric ROX to identify HFNC failure in children with different etiologies of respiratory diseases; however, they used the respiratory z score, which is relatively complicated to apply at the bedside. Subsequently, two other studies used the ROX index in the pediatric population, but only one of them was specific to infants with bronchiolitis^16,17^. Our hypothesis is that the ROX index is a promising predictor of the need to escalate ventilatory support from HFNC to NIV/IMV.

## Patients and methods

### Study type and location

A prospective, observational, multicenter clinical study was conducted in 2 ICUs, one of which was a quaternary-level private hospital and the other of which was a tertiary-level public hospital, from September 2020 to July 2022. Both ICUs are exclusively pediatric and admit patients aged 0–17 years and 11 months. This study followed the recommendations of the “Strengthening the Reporting of Observational Studies in Epidemiology” (STROBE) guidelines for observational studies.

### Inclusion criteria

Infants under 2 years old diagnosed with bronchiolitis and treated with HFNC therapy were included.

### Exclusion criteria

Patients who used HFNC therapy for more than 2 h before inclusion in the study, patients with heart disease, patients with major malformations (anatomical upper airway obstruction) and infants whose parents did not agree to participate in the study were excluded.

### Ethical aspects

The study was approved by the Research Ethics Committee of Israelita Albert Einstein Hospital, and the parents or legal guardians of the infants signed the informed consent form. This study is in accordance with the recently amended Declaration of Helsinki of 1975. The risk of participating in this study was considered minimal and was limited by the accidental loss of confidentiality of the collected data.

### Protocol

Clinical variables and HFNC settings were collected immediately before the initiation of HFNC therapy, at 2, 6-, 12-, 18-, and 24-h post-initiation, and at the time of HFNC removal. A variation of 15 min earlier or later was allowed for each assessment. The main outcome was failure of HFNC support, which was defined as the need for a change to NIV or IMV support. Failure was determined by the treating team but was typically the result of refractory tachypnea, respiratory distress, and/or hypoxemia.

### Clinical evaluation

Clinical parameters, such as heart rate (HR), respiratory distress score, respiratory rate (RR), fraction of inspired oxygen (FiO_2_) and oxygen saturation (SpO_2_), were collected by four respiratory therapists from the assistance team at just prior to HFNC initiation; 2, 6, 12, 18, and 24 h after HFNC initiation; and at the time of removal from HFNC. SpO_2_, FiO_2_ and RR will be used to calculate the ROX index^10^. We calculated the S/F as the SpO_2_ (%) divided by the FIO_2_. ROX was calculated as S/F divided by RR.

$${\text{S}}/{\text{F}} = \frac{{{\text{SpO}}_{2} }}{{{\text{FiO}}_{2} }} \, \left( \% \right)$$

$${\text{ROX}} = \frac{{{\text{S}}/{\text{F}}}}{{{\text{RR}}}}$$

To assess respiratory distress, the respiratory distress score described by Ben-Zvi^18,19^, which assesses auscultation and the use of accessory muscles and ranges from 0 to 7 points, was used. At our institution, we considered patients who were indicated for the use of HFNC therapy to have a respiratory distress score between 2 and 4. RR was assessed by counting the number of cycles for 1 min. Heart rate (HR) and SpO_2_ were obtained directly from the Infinity Delta multiparameter monitor (Dräger, Lubeck, Germany).

The risk of mortality was assessed using the Pediatric Index of Mortality (PIM2) score collected during the first 24 h of hospitalization^20^. Furthermore, outcomes such as the duration of respiratory support (considered the period of use of HFNC therapy, NIV/IMV and conventional oxygen therapy), length of stay in the ICU and length of stay in the hospital were also evaluated.

### High flow equipment

One of the two systems available in pediatric ICUs was used to perform HFNC therapy: Precision Flow (Vapotherm, New Hampshire, USA) and Optflow (Fisher & Paykel Healthcare, Auckland—New Zealand, MR850 humidification system, RT330 Kit). Optiflow oxygen cannulas).

### Statistical analysis

Data were described using absolute and relative frequencies for categorical variables and as medians and quartiles, in addition to minimum and maximum values for numerical variables. The distributions of the numerical variables were studied using histograms, boxplots, and the Shapiro‒Wilk normality test^21^. Nonparametric Mann‒Whitney tests were used for the numerical variables to compare the groups with HFNC failure and success in terms of baseline and late outcome data.

The clinical parameters were compared between the HFNC failure and success groups at each evaluation time point using generalized mixed models or generalized linear models^22^ considering the best fit and the dependence between the evaluations performed on the same child. The results of the models were presented as the mean values estimated by the models, 95% confidence intervals (CI) and p values corrected by the sequential Bonferroni method, considering a significance level of 5%.

The performance of the ROX index, S/F ratio and respiratory distress score at the different evaluation times in the discrimination of patients with and without HFNC failure was investigated by receiver operating characteristic (ROC) curves, and the best cutoff point was determined based on the Youden index^23^.

The analyses were performed using the SPSS^24^ and R^25^ statistical packages, considering a significance level of 5%.

## Results

A total of 102 infants were included in the study. The demographic characteristics are shown in Table 1. There was no evidence of differences between the success and failure groups in HFNC therapy regarding the demographic characteristics of the patients (*p* > 0.05 in all comparisons). No difference was observed between the type of equipment used and therapy failure. There were 84 (82.4%) successful patients and 18 (17.6%) failures in HFNC therapy. Among the patients who failed, 15 (83.3%) patients required NIV, and four (22.2%) required IMV, one of whom was placed on NIV and one on IMV.

**Table 1:** Demographic and clinical characteristics of infants with bronchiolitis using high-flow nasal cannula.

	Total (n = 102)	Outcome		*p* value
		Failure (n = 18)	Success (n = 84)	
Age (months)				0.975^#^
Median (Q1; Q3)	3.4 (1.6; 6.5)	2.9 (2.1; 6.7)	3.5 (1.3; 6.5)	
Weight (kg)				0.857^#^
Median (Q1; Q3)	5.6 (4.4; 7.3)	5.8 (4.3; 7.0)	5.5 (4.5; 7.3)	
PIM2				0.979^#^
Median (Q1; Q3)	0.59 (0.20; 0.60)	0.40 (0.20; 0.60)	0.59 (0.21; 0.60)	

PIM 2, pediatric index of mortality; HFNC, high flow nasal cannula.

Q1: first quartile; Q3: third quartile; n: number of patients; #Mann‒Whitney test.

**Figure 1:**
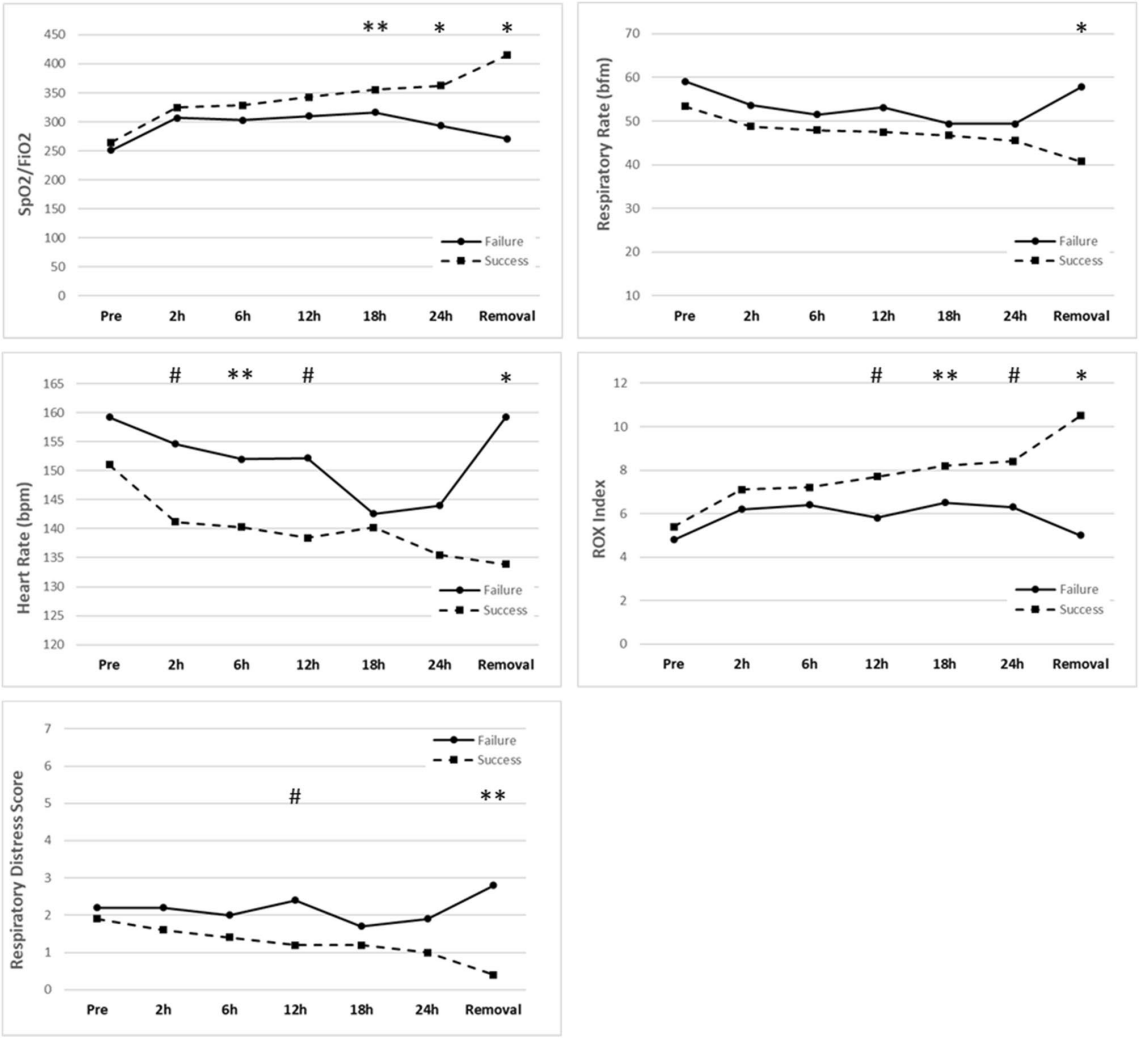
Evolution of physiological variables, including the ROX index, during the 7 evaluation times and the differentiation between patients who had failure (solid line) and success (dashed line) in HFNC therapy. *p* values are only presented when there is a significant difference between the groups, and the absence of mention indicates no significant difference. **p < 0.05, #*p* < 0.01, *p < 0.001. The values are expressed as estimated means and 95% confidence intervals (95% CI) obtained through generalized mixed models (moments from before to 24 h) and generalized linear models (removal moment. The removal time was adjusted for the time of use of HFNC therapy.

The comparisons between the groups that had failure or success in relation to the clinical parameters at each evaluation are described in Table 2 and Fig. 1. For the ROX index, significant differences were observed between the failure 5.8 (95% CI 4.7–7.1) and success 7.7 (95% CI 7.2–8.2) groups (*p* = 0.005) from the 12-h evaluation. We also observed significant differences at 18 and 24 h for both the ROX index and the S/F ratio. The respiratory distress score differed between the groups only at the 12-h evaluation, as shown in Table 2 and Fig. 1. There was no significant difference in HR after2 hours, and there was no evidence of differences between the failure and success groups regarding the mean respiratory rate at most evaluations except at the time of removal (Fig. 1).

**Table 2:** Estimated mean values and 95% confidence intervals for clinical parameters in infant patients with bronchiolitis using high-flow nasal cannula.

		Outcome		Estimated mean differences	
		Failure (n = 18)	Success (n = 84)	Success–Failure	*p* value
ROX index	n observed				
Pre	8/31	4.8 (3.6; 6.4)	5.4 (4.7; 6.1)	0.6 (-1.0; 2.1)	0.460
2 h	17/84	6.2 (5,2; 7,4)	7,1 (6,6; 7,7)	1,0 (-0,2; 2,2)	0,118
6 h	16/84	6.4 (5.3; 7.6)	7.2 (6.7; 7.8)	0.9 (-0.4; 2.1)	0.178
12 h	12/82	5.8 (4.7; 7.1)	7.7 (7.2; 8.2)	1.9 (0.6; 3.3)	0.005
18 h	8/82	6.5 (5.2; 8.2)	8.2 (7.7; 8.8)	1.7 (0.1; 3.2)	0.033
24 h	9/80	6.3 (5.0; 8.0)	8.4 (7.9; 9.0)	2.1 (0.5; 3.6)	0.009
Removal	16/83	5.0 (4.0; 6.4)	10.5 (10.0; 11.1)	5.5 (4.2; 6.8)	< 0.001
S/F ratio	n observed				
Pre	8/31	251.1 (176.8; 356.6)	264.6 (221.7; 315.8)	13.5 (-86.2; 113.3)	0.790
2 h	17/84	306.5 (275.8; 340.6)	324.4 (309.3; 340.2)	17.9 (-18.0; 53.7)	0.328
6 h	16/84	303.0 (271.2; 338.4)	328.7 (313.0; 345.1)	25.7 (-11.5; 62.9)	0.175
12 h	12/82	309.7 (280.8; 341.6)	342.9 (329.2; 357.3)	33.2 (-0.2; 66.7)	0.051
18 h	8/82	316.4 (283.5; 353.0)	355.1 (340.6; 370.3)	38.7 (1.0; 76.5)	0.044
24 h	9/80	293.0 (258.5; 332.2)	362.7 (345.9; 380.3)	69.6 (29.0; 110.3)	0.001
Removal	16/83	270.7 (247.6; 295.9)	414.9 (399.0; 431.4)	144.2 (115.2; 173.3)	< 0.001
Respiratory distress score	n observed				
Pre	17/81	2.2 (1.6; 2.9)	1.9 (1.7; 2.2)	-0.2 (-0.9; 0.5)	0.547
2 h	17/81	2.2 (1.7; 2.9)	1.6 (1.4; 1.8)	-0.6 (-1.3; 0.0)	0.056
6 h	15/77	2.0 (1.5; 2.7)	1.4 (1.2; 1.6)	-0.6 (-1.3; 0.0)	0.056
12 h	10/75	2.4 (1.7; 3.3)	1.2 (1.1; 1.4)	-1.1 (-1.9; -0.3)	0.006
18 h	5/70	1.7 (1.0; 2.8)	1.2 (1.0; 1.4)	-0.5 (-1.4; 0.3)	0.241
24 h	6/68	1.9 (1.0; 3.4)	1.0 (0.8; 1.3)	-0.9 (-2.0; 0.3)	0.135
Removal	9/62	2.8 (1.2; 6.4)	0.4 (0.3; 0.7)	-2.4 (-4.7; 0.0)	0.049

Values expressed as estimated means and 95% confidence intervals (95% CI) obtained through generalized mixed models (moments from before to 24 h) and generalized linear models (removal moment);

In mixed models, the *p* values presented were corrected by the sequential Bonferroni method.

The removal time was adjusted for the time of HFNC use.

The low number of patients evaluated in the pre moment in relation to S/F and ROX index is due to the oxygen therapy device used, which does not provide a reliable FiO_2_ but is adjusted with oxygen flow.

**Figure 2:**
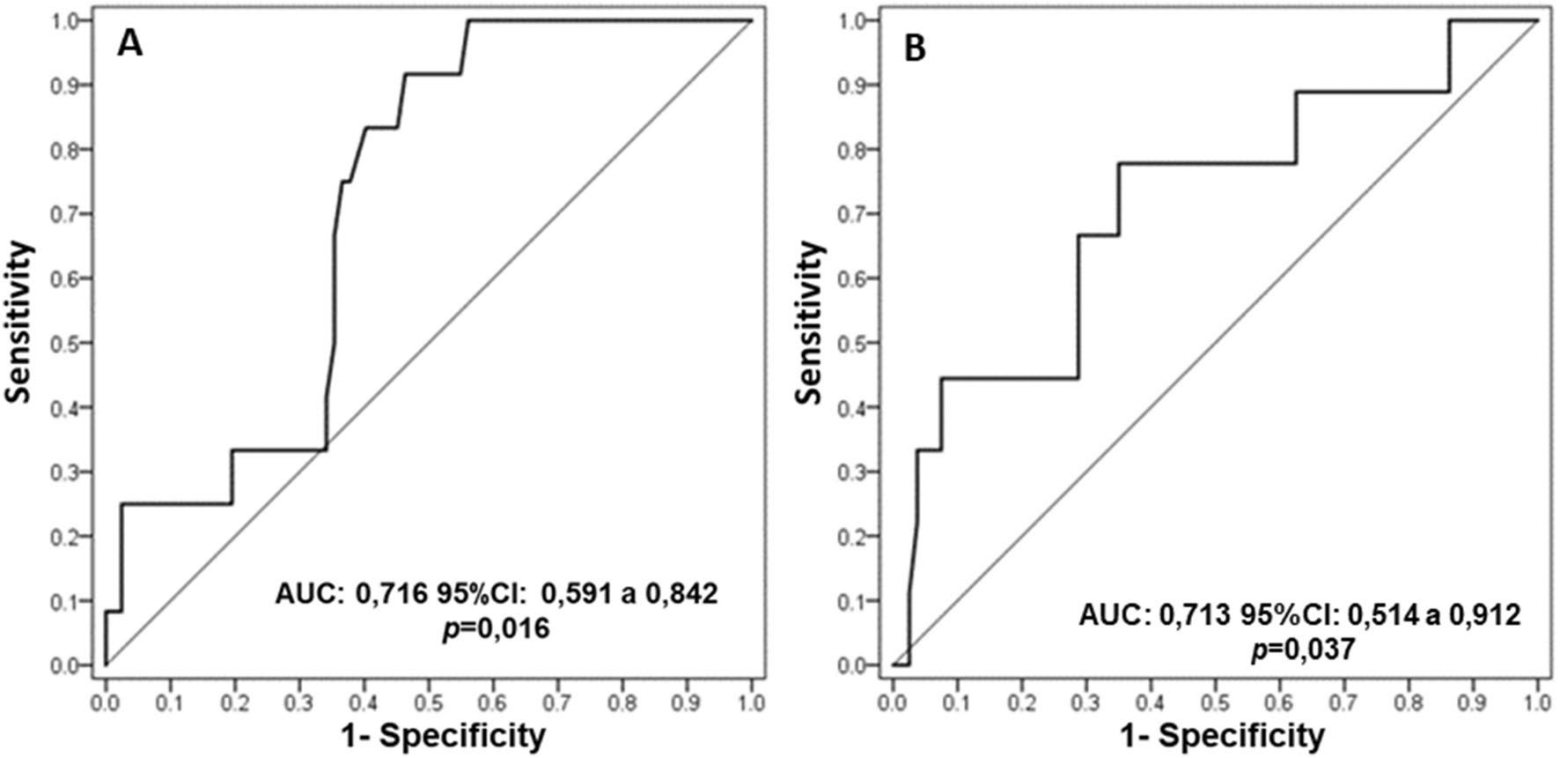
ROC curves for the ROX index in predicting failure of high-flow nasal cannula therapy in infants with bronchiolitis. Figure (**A**) corresponds to the AUC for the 12-h time point, and Figure (**B**) corresponds to the AUC for the 24-h time point.

We investigated the performance of the ROX index at each evaluation time point in the discrimination of patients with success from those with failure by means of ROC curves (Fig. 2). We observed areas under the curve (AUCs) significantly greater than 0.500 at 12 h, with a value of 0.716 (95% CI 0.591–0.842; *p* = 0.016), and at 24 h, with values of 0.713 (95% CI 0.514–0.912; *p* = 0.037) for the ROX index.

We also calculated the ROC curves of the S/F ratio and the respiratory distress score at 12 h, and the ROX index values for these other variables are presented in Table 3.

**Table 3:** ROC curves for ROX, S/F ratio and respiratory distress score in the discrimination of failure and success in infant patients with bronchiolitis using high-flow nasal cannula.

	Failure/Success	AUC	95% CI	*p* value
ROX index				
12 h	12/82	0.716	0.59; 0.84	0.016
S/F ratio				
12 h	12/82	0.629	0.46; 0.79	0.150
Respiratory distress score				
12 h	12/82	0.713	0.51; 0,85	0.027

AUC, area under the ROC curve; 95% CI, 95% confidence interval for AUC.

The best cutoff range found in our study was 6.50–7.18 points for the ROX index at 12 h, with sensitivity 42%, specificity 66%, negative predictive value (NPV) 15% positive predictive value (PPV) 89% for 6.50 and sensitivity 92%, specificity 54%, negative predictive value (NPV) 22% positive predictive value (PPV) 98% for 7.18 and for respiratory distress score the best cutoff point was 1.5 at 12 h with sensitivity 80% and specificity 59% (Table 4). The complete table of sensitivity, specificity, NPV and PPV values for the ROX index is available in the supplementary materials.

**Table 4:** Best cutoff range for the ROX index and respiratory distress score at 12 h.

Predictors	Cutoff value 12 h	Sensitivity (%)	Specificiy (%)	NPV (%)	PPV (%)
ROX index	5.56	33	80	20	89
	6.50	42	66	15	89
	6.82	83	60	23	96
	7.18	92	54	22	98
	7.95	100	44	21	100
	8.52	100	34	18	100
Respiratoy distress score	0.5	100	13	13	100
	1.5	80	59	21	96
	2.5	20	92	25	90

NPV, negative predictive value; PPV, positive predictive value.

For the late outcomes, we found evidence of differences between the failure and success groups (Table 5) in relation to the time of use of HFNC therapy (*p* = 0.008), duration of respiratory support (*p* < 0.001), length of stay in the ICU (*p* < 0.001) and length of hospital stay (*p* < 0.001). We observed that patients in the successful group had a longer median time of use of HFNC therapy and shorter median duration of respiratory support, length of stay in the ICU and length of hospital stay than patients in the failure group did.

**Table 5:** Late outcome of infant patients with bronchiolitis using high-flow nasal cannula.

	Total (n = 102)	Outcome		*p* value
		Failure (n = 18)	Success (n = 84)	
Time of use of HFNC (hours)				0.008^#^
Median (Q1; Q3)	57.0 (37.3; 81.5)	22.3 (8.0; 76.0)	63.0 (41.4; 82.2)	
Duration of respiratory support (hours)				< 0.001^#^
Median (Q1; Q3)	87.3 (50.8; 134.1)	135.0 (98.5; 209.1)	78.7 (42.5; 116.7)	
Length of ICU (days)				< 0.001^#^
Median (Q1; Q3)	4.6 (3.6; 6.5)	7.5 (5.0; 12.0)	4.3 (3.4; 5.6)	
Length of hospital stay (days)				< 0.001^#^
Median (Q1; Q3)	6.0 (4.1; 8.0)	9.1 (6.4; 13.3)	5.2 (4.0; 7.5)	

HFNC, high flow nasal cannula; ICU, intensive care unit.

Time of use of HFNC (hours): time of use of HFNC from installation until removal (installation of conventional oxygen therapy or room air in the success group and installation of NIV or IMV in the failure group.

Duration of respiratory support (considered the period of use in the HFNC, NIV/IMV and conventional oxygen therapy).

Q1: first quartile; Q3: third quartile; n: number of patients; #Mann‒Whitney tests.

## Discussion

Our study including 102 infants showed that the ROX index is effective at predicting failure of HFNC therapy in infants with bronchiolitis from 12 h after onset, with an area under the curve of 0.716 (*p* = 0.016). We considered the value for the best cutoff point based on the Youden index, which is considered the best cutoff point for which the sum of sensitivity and specificity reaches the highest value; however, the best cutoff depends on the system and resources available, and we encouraged the reader to evaluate the other cutoff ranges presented. Instead of focusing on a cutoff point, we can work with a cutoff range for the ROX index. The best cutoff range found in our study was 6.50–7.18 points, with a sensitivity of 42%, specificity of 66%, NPV of 15% and PPV of 89% for 6.50 and a sensitivity of 92%, specificity of 54%, NPV of 22% and PPV of 98% for 7.18; these findings could guide its use in clinical practice. In addition to the ROX, we also evaluated other physiological variables and their evolution over time, expanding the interpretation of these data.

The literature has evaluated clinical signs such as SpO_2_, FiO_2_, the S/F ratio, the respiratory rate, and respiratory distress alone, but a consensus has not been reached. While some authors^8,26^ highlighted respiratory rate, FiO_2_ and SpO_2_ in the successful group in the treatment of HFNC in the first hours, others^9,27^ showed variables such as HR and initial respiratory acidosis as significant predictors of HFNC failure.

Therefore, identifying associations between vital signs seems to be sensible for improving accuracy. Roca et al. developed and validated a new index based on oxygenation and the RR in adult patients; this index showed good accuracy as a predictor of the need for IMV in adult patients, with a cutoff value of 4.88 at 12 h^10^. Our study found similar results in the pediatric population, performing similar analyses. However, because the RR in infants is quite different from that in adults, the ROX value in our study was 7.18 at 12 h after installation.

During the course of our study, 3 studies were published using the ROX for the pediatric population^16,17,28^. Yildizdas et al.^28^ and Kim et al.^16^ were the first authors to publish the ROX for the pediatric population; however, these studies included patients aged 0–18 years, and due to the great variability of RR in this age range, it was necessary to modulate RR with the Z score, thus changing the index value. Yildizdas et al. showed that the p-ROXI (pediatric respiratory rate-oxygenation index) can be used as a good marker for predicting the risk of HFNC failure in patients with acute respiratory failure. These results reinforce the use of the ROX in the pediatric population; however, the analysis methods used in these studies make its application at the bedside difficult.

To the best of our knowledge, only one study other than ours, which included only infants, has used the ROX score to predict HFNC failure^17^. Kannikeswaran et al.^17^ assessed 373 infants with bronchiolitis and found that patients who required positive pressure ventilation had a lower ROX score compared to those who did not (5.86; 4.71–7.42 vs. 6.74; 5.46–8.25; *p* = 0.01). Additionally, patients with a ROX index below 5.39 were three times more likely to need positive pressure ventilation than those with a ROX index higher than 8.21. In our study, we observed that a ROX of 5.56 at 12 h was associated with a sensitivity of 33%. This indicates that very low cutoff values may underestimate the need for escalating ventilatory support in treated patients. On the other hand, excessively high cutoff values can lead to inappropriate resource utilization. Therefore, we chose to present our results as a cutoff range instead of a specific cutoff point. This approach provides greater flexibility to the healthcare team during the decision-making process, ensuring a more precise adaptation to individual patient needs and other follow-up; this is a limitation of this study.

Our study evaluated the ROX score and other clinical variables in infants at 7 time points during the first 24 h of HFNC therapy: before admission; after 2, 6, 12, 18 and 24 h; and until removal. This follow-up with short assessment intervals allowed us to observe interesting data on the evolution of these patients during HFNC therapy, such as the improvement in the first 6 h, regardless of whether they had failed the treatment. In contrast to what occurs with the use of NIV, in which a difference is observed between responders and nonresponders in the first 2 h^29–31^, patients receiving HFNC therapy seem to experience a slightly different evolution. Er et al.^27^ reported similar results, showing improvement in clinical signs in the first 4 h in both the success and failure groups. Furthermore, other authors also described that failure occurred between 5 and 12 h after starting therapy^7,30,32,33^. This finding reinforces the importance of requiring constant clinical assessment throughout therapy.

Returning to the importance of the association of clinical variables, if we look at our results for the S/F ratio and RR alone, the evaluation of RR showed no difference between the infants with success or failure at any time, and the S/F ratio was only significant after 18 h. By associating the two variables using the ROX, it was possible to differentiate patients from 12 h onward. In addition, the ROC curve for the S/F ratio was greater than 0.7 only at 24 h, which is later than the ROX index, suggesting once again that the use of ROX is encouraged in clinical practice.

However, when we evaluated the respiratory distress score, the ROC curve was similar to that of the ROX index, with an AUC of 0.71 and a PPV of 96%. Saelim et al. evaluated the performance of the pROX index and clinical respiratory score (CRS) (in our study, the Ben-Zvi score was used) for predicting HFNC-related outcomes and reported that the discomfort score was superior to the ROX index, with an AUC of 0.92 versus 0.77 for the index pROX. This study included 1-month-old to 15-year-old patients diagnosed with respiratory distress syndrome, and due to age variability, the RR had to be adjusted. Despite the good results found in both our study and Saelim's study in the use of the respiratory distress score for predicting HFNC failure, the difficulty of using respiratory distress assessment instruments must be considered. There is great variability in the instruments that have been used without proper validation for the target population to which the instrument will be applied, in addition to the need for translation into the language of the country that intends to use the instrument.

Adopting the ROX value of 6.50–7.18 as the best cutoff presented the best sensitivity and specificity at 12 h, worsening at 18 and 24 h. In contrast to the results of Roca et al.^10^, a cutoff value of 4.8 at 12 h was associated with improved accuracy at 18 and 24 h. This can be explained by the small number of patients in the failure group at 18 and 24 h.

The assessment of HR alone in our study showed a significant difference from 2 h onward. Some authors have proposed incorporating the HR into the ROX index, the ROX-HR; however, the results have not shown superiority in relation to the ROX index^34,35^. In addition to the lack of superiority, a factor that may confound the evaluation of HR is the variation caused by the use of bronchodilators, which often causes an increase in HR as a side effect. Therefore, the increase in HR may be influenced by the action of the drug and not by an increase in metabolic demand.

Regarding late clinical outcomes, longer stays in the ICU and hospital were observed in infants who failed HFNC therapy, as was the case in other studies^27,33^. Although the time of use of HFNC therapy was longer in the success group, the total duration of respiratory support was much shorter, which was likely influenced by the length of hospital stay. This result reinforces the need to broaden our understanding of the factors of therapy failure to advance in the form of prevention, thus reflecting a decrease in hospitalization time and, consequently, cost.

Our study has several limitations. First, our findings were obtained in a cohort of limited size. The second limitation is the small sample size in the failure group. Although we included a total of 102 patients, our low therapy failure rate, with only 18 patients failing in the group, may limit the interpretability of our data, especially in terms of the variables or in the moments in which no differences were detected. The third limitation is that we lost data on the respiratory distress score throughout the evaluation period, but mainly at the time of HFNC therapy removal. Fourth, the inclusion of only infants with bronchiolitis may limit the generalization of the data; on the other hand, in children in this age group, this will be the main pathological condition.

## Conclusion

The ROX index appears effective in predicting HFNC therapy failure in infants with bronchiolitis at 12 h evaluation. A cutoff range of 6.50–7.18 points, suggested by our study, may serve as a practical guideline. For institutions with more limited resources, the suggestion to use cutoff points closer to 6.50 on the ROX index seems prudent, as this can help avoid unnecessary escalation of ventilatory support. On the other hand, institutions with a more complex patient profile can consider cutoff points closer to 7.18, which demonstrate greater sensitivity, reducing the risk of not indicating the escalation of support for more critical patients. This personalized approach based on the institution's characteristics and resources is a relevant and practical conclusion, contributing to the clinical application of the results. Nevertheless, due to the limited cohort size, further validation on a larger scale is essential.

### Supplementary Information

Supplementary Tables.

Supplementary Information 2.

## Data Availability

All the data generated or analyzed during this study are included in this published article (and its Supplementary Information files). The datasets generated and/or analyzed during the current study are available from the corresponding author upon reasonable request.
