# Supplementary material for: ROX index as a predictor of failure of high-flow nasal cannula in infants with bronchiolitis
Source: Sci Rep. 2024 Jan 3;14:389. doi: 10.1038/s41598-024-51214-4 (PMC10764845; doi:10.1038/s41598-024-51214-4)
Supplement: Supplementary file 1 — Supplementary Tables. [file 41598_2024_51214_MOESM1_ESM.docx]

Appendix 1 - ROC curves for ROX index and S/F ratio (SpO2/FIO2) in the discrimination of patients without and with failure

| Table I - ROC curves for the ROX index in relation to therapy failure in infant patients with bronchiolitis using a high-flow nasal cannula | | | |
| --- | --- | --- | --- |
| ROX index | Sensitivity | 1 - Specificity | Specificity |
| **2 hours** |  |  |  |
| 1.430 | 0.0000 | 0.0000 | 1.0000 |
| 2.675 | 0.0588 | 0.0000 | 1.0000 |
| 3.030 | 0.0588 | 0.0119 | 0.9881 |
| 3.320 | 0.1176 | 0.0119 | 0.9881 |
| 3.595 | 0.1176 | 0.0357 | 0.9643 |
| 3.735 | 0.1176 | 0.0476 | 0.9524 |
| 3.845 | 0.1176 | 0.0595 | 0.9405 |
| 3.930 | 0.1765 | 0.0595 | 0.9405 |
| 3.975 | 0.1765 | 0.0714 | 0.9286 |
| 4.080 | 0.1765 | 0.0833 | 0.9167 |
| 4.270 | 0.2353 | 0.0833 | 0.9167 |
| 4.410 | 0.2353 | 0.0952 | 0.9048 |
| 4.450 | 0.2353 | 0.1071 | 0.8929 |
| 4.480 | 0.2353 | 0.1190 | 0.8810 |
| 4.520 | 0.2353 | 0.1429 | 0.8571 |
| 4.595 | 0.2353 | 0.1548 | 0.8452 |
| 4.700 | 0.2353 | 0.1667 | 0.8333 |
| 4.765 | 0.2941 | 0.1667 | 0.8333 |
| 4.790 | 0.2941 | 0.1786 | 0.8214 |
| 4.815 | 0.2941 | 0.1905 | 0.8095 |
| 4.835 | 0.2941 | 0.2024 | 0.7976 |
| 4.870 | 0.2941 | 0.2143 | 0.7857 |
| 4.940 | 0.2941 | 0.2262 | 0.7738 |
| 5.015 | 0.2941 | 0.2381 | 0.7619 |
| 5.060 | 0.2941 | 0.2500 | 0.7500 |
| 5.080 | 0.3529 | 0.2500 | 0.7500 |
| 5.095 | 0.3529 | 0.2619 | 0.7381 |
| 5.115 | 0.3529 | 0.2738 | 0.7262 |
| 5.145 | 0.3529 | 0.2857 | 0.7143 |
| 5.190 | 0.4118 | 0.2857 | 0.7143 |
| 5.255 | 0.4118 | 0.2976 | 0.7024 |
| 5.330 | 0.4118 | 0.3095 | 0.6905 |
| 5.380 | 0.4118 | 0.3214 | 0.6786 |
| 5.465 | 0.4118 | 0.3333 | 0.6667 |
| 5.555 | 0.4118 | 0.3452 | 0.6548 |
| 5.585 | 0.4118 | 0.3571 | 0.6429 |
| 5.665 | 0.4118 | 0.3690 | 0.6310 |
| 5.750 | 0.4706 | 0.3690 | 0.6310 |
| 5.800 | 0.4706 | 0.3810 | 0.6190 |
| 5.890 | 0.5294 | 0.3810 | 0.6190 |
| 5.975 | 0.5294 | 0.3929 | 0.6071 |
| 6.025 | 0.5294 | 0.4048 | 0.5952 |
| 6.260 | 0.5294 | 0.4167 | 0.5833 |
| 6.500 | 0.5294 | 0.4286 | 0.5714 |
| 6.565 | 0.5294 | 0.4405 | 0.5595 |
| 6.610 | 0.5294 | 0.4524 | 0.5476 |
| 6.645 | 0.5294 | 0.4643 | 0.5357 |
| 6.690 | 0.5294 | 0.4762 | 0.5238 |
| 6.725 | 0.5294 | 0.4881 | 0.5119 |
| 6.765 | 0.5882 | 0.5000 | 0.5000 |
| 6.840 | 0.6471 | 0.5000 | 0.5000 |
| 7.000 | 0.6471 | 0.5119 | 0.4881 |
| 7.140 | 0.6471 | 0.5238 | 0.4762 |
| 7.180 | 0.6471 | 0.5357 | 0.4643 |
| 7.230 | 0.6471 | 0.5476 | 0.4524 |
| 7.300 | 0.6471 | 0.5595 | 0.4405 |
| 7.580 | 0.6471 | 0.5714 | 0.4286 |
| 7.835 | 0.6471 | 0.5833 | 0.4167 |
| 7.865 * | 0.7647 | 0.5833 | 0.4167 |
| 7.945 | 0.7647 | 0.6071 | 0.3929 |
| 8.040 | 0.7647 | 0.6429 | 0.3571 |
| 8.125 | 0.7647 | 0.6786 | 0.3214 |
| 8.185 | 0.7647 | 0.7024 | 0.2976 |
| 8.225 | 0.8235 | 0.7024 | 0.2976 |
| 8.335 | 0.8235 | 0.7143 | 0.2857 |
| 8.460 | 0.8235 | 0.7262 | 0.2738 |
| 8.505 | 0.8235 | 0.7381 | 0.2619 |
| 8.520 | 0.8824 | 0.7381 | 0.2619 |
| 8.570 | 0.8824 | 0.7500 | 0.2500 |
| 8.625 | 0.8824 | 0.7619 | 0.2381 |
| 8.685 | 0.8824 | 0.7857 | 0.2143 |
| 8.805 | 0.8824 | 0.7976 | 0.2024 |
| 8.920 | 0.8824 | 0.8095 | 0.1905 |
| 8.980 | 0.9412 | 0.8095 | 0.1905 |
| 9.120 | 0.9412 | 0.8214 | 0.1786 |
| 9.285 | 1.0000 | 0.8214 | 0.1786 |
| 9.475 | 1.0000 | 0.8333 | 0.1667 |
| 9.660 | 1.0000 | 0.8690 | 0.1310 |
| 9.750 | 1.0000 | 0.8810 | 0.1190 |
| 9.850 | 1.0000 | 0.8929 | 0.1071 |
| 10.080 | 1.0000 | 0.9048 | 0.0952 |
| 10.350 | 1.0000 | 0.9167 | 0.0833 |
| 10.765 | 1.0000 | 0.9286 | 0.0714 |
| 11.320 | 1.0000 | 0.9405 | 0.0595 |
| 11.840 | 1.0000 | 0.9524 | 0.0476 |
| 12.255 | 1.0000 | 0.9643 | 0.0357 |
| 12.925 | 1.0000 | 0.9762 | 0.0238 |
| 13.805 | 1.0000 | 0.9881 | 0.0119 |
| 15.140 | 1.0000 | 1.0000 | 0.0000 |
|  |  |  |  |
| **6 hours** |  |  |  |
| 2.230 | 0.0000 | 0.0000 | 1.0000 |
| 3.300 | 0.0625 | 0.0119 | 0.9881 |
| 3.395 | 0.0625 | 0.0238 | 0.9762 |
| 3.465 | 0.1250 | 0.0238 | 0.9762 |
| 3.635 | 0.1250 | 0.0357 | 0.9643 |
| 3.815 | 0.1250 | 0.0476 | 0.9524 |
| 3.955 | 0.1875 | 0.0476 | 0.9524 |
| 4.085 | 0.1875 | 0.0714 | 0.9286 |
| 4.135 | 0.1875 | 0.0833 | 0.9167 |
| 4.165 | 0.1875 | 0.0952 | 0.9048 |
| 4.205 | 0.2500 | 0.0952 | 0.9048 |
| 4.235 | 0.2500 | 0.1071 | 0.8929 |
| 4.270 | 0.2500 | 0.1190 | 0.8810 |
| 4.350 | 0.2500 | 0.1310 | 0.8690 |
| 4.480 | 0.3125 | 0.1310 | 0.8690 |
| 4.665 | 0.3125 | 0.1429 | 0.8571 |
| 4.810 | 0.3125 | 0.1548 | 0.8452 |
| 4.870 | 0.3125 | 0.1667 | 0.8333 |
| 4.925 | 0.3125 | 0.1786 | 0.8214 |
| 5.000 | 0.3125 | 0.1905 | 0.8095 |
| 5.065 | 0.3125 | 0.2024 | 0.7976 |
| 5.085 | 0.3125 | 0.2143 | 0.7857 |
| 5.155 | 0.3125 | 0.2262 | 0.7738 |
| 5.245 | 0.3125 | 0.2381 | 0.7619 |
| 5.285 | 0.3125 | 0.2500 | 0.7500 |
| 5.345 | 0.3750 | 0.2619 | 0.7381 |
| 5.415 | 0.4375 | 0.2619 | 0.7381 |
| 5.515 | 0.4375 | 0.2857 | 0.7143 |
| 5.620 | 0.5000 | 0.2857 | 0.7143 |
| 5.655 | 0.5000 | 0.2976 | 0.7024 |
| 5.745 | 0.5000 | 0.3095 | 0.6905 |
| 5.855 | 0.5000 | 0.3214 | 0.6786 |
| 5.920 | 0.5625 | 0.3214 | 0.6786 |
| 5.980 | 0.5625 | 0.3333 | 0.6667 |
| 6.045 | 0.5625 | 0.3452 | 0.6548 |
| 6.160 | 0.5625 | 0.3571 | 0.6429 |
| 6.240 * | 0.6250 | 0.3571 | 0.6429 |
| 6.390 | 0.6250 | 0.3690 | 0.6310 |
| 6.565 | 0.6250 | 0.3810 | 0.6190 |
| 6.635 | 0.6250 | 0.3929 | 0.6071 |
| 6.700 | 0.6250 | 0.4048 | 0.5952 |
| 6.770 | 0.6250 | 0.4167 | 0.5833 |
| 6.830 | 0.6250 | 0.4286 | 0.5714 |
| 6.865 | 0.6250 | 0.4405 | 0.5595 |
| 6.930 | 0.6250 | 0.4524 | 0.5476 |
| 6.990 | 0.6250 | 0.4643 | 0.5357 |
| 7.050 | 0.6250 | 0.4762 | 0.5238 |
| 7.205 | 0.6250 | 0.5000 | 0.5000 |
| 7.345 | 0.6250 | 0.5119 | 0.4881 |
| 7.435 | 0.6250 | 0.5238 | 0.4762 |
| 7.495 | 0.6250 | 0.5476 | 0.4524 |
| 7.515 | 0.6250 | 0.5595 | 0.4405 |
| 7.535 | 0.6875 | 0.5595 | 0.4405 |
| 7.570 | 0.6875 | 0.5833 | 0.4167 |
| 7.610 | 0.6875 | 0.5952 | 0.4048 |
| 7.645 | 0.6875 | 0.6071 | 0.3929 |
| 7.675 | 0.6875 | 0.6190 | 0.3810 |
| 7.720 | 0.6875 | 0.6310 | 0.3690 |
| 7.810 | 0.6875 | 0.6429 | 0.3571 |
| 8.060 | 0.7500 | 0.6548 | 0.3452 |
| 8.305 | 0.7500 | 0.6667 | 0.3333 |
| 8.390 | 0.7500 | 0.6786 | 0.3214 |
| 8.435 | 0.7500 | 0.6905 | 0.3095 |
| 8.480 | 0.7500 | 0.7024 | 0.2976 |
| 8.535 | 0.7500 | 0.7143 | 0.2857 |
| 8.630 | 0.7500 | 0.7262 | 0.2738 |
| 8.760 | 0.7500 | 0.7381 | 0.2619 |
| 8.870 | 0.7500 | 0.7500 | 0.2500 |
| 8.955 | 0.7500 | 0.7619 | 0.2381 |
| 8.990 | 0.7500 | 0.7738 | 0.2262 |
| 9.010 | 0.7500 | 0.7857 | 0.2143 |
| 9.045 | 0.7500 | 0.7976 | 0.2024 |
| 9.095 | 0.7500 | 0.8095 | 0.1905 |
| 9.130 | 0.7500 | 0.8214 | 0.1786 |
| 9.150 | 0.8125 | 0.8333 | 0.1667 |
| 9.200 | 0.8750 | 0.8333 | 0.1667 |
| 9.305 | 0.8750 | 0.8452 | 0.1548 |
| 9.425 | 0.9375 | 0.8452 | 0.1548 |
| 9.600 | 1.0000 | 0.8452 | 0.1548 |
| 9.830 | 1.0000 | 0.8571 | 0.1429 |
| 9.970 | 1.0000 | 0.8690 | 0.1310 |
| 10.015 | 1.0000 | 0.8810 | 0.1190 |
| 10.090 | 1.0000 | 0.8929 | 0.1071 |
| 10.155 | 1.0000 | 0.9048 | 0.0952 |
| 10.185 | 1.0000 | 0.9167 | 0.0833 |
| 10.290 | 1.0000 | 0.9286 | 0.0714 |
| 10.630 | 1.0000 | 0.9405 | 0.0595 |
| 10.945 | 1.0000 | 0.9524 | 0.0476 |
| 11.275 | 1.0000 | 0.9643 | 0.0357 |
| 12.170 | 1.0000 | 0.9762 | 0.0238 |
| 13.610 | 1.0000 | 0.9881 | 0.0119 |
| 15.430 | 1.0000 | 1.0000 | 0.0000 |
|  |  |  |  |
| **12 hours** |  |  |  |
| 2.370 | 0.0000 | 0.0000 | 1.0000 |
| 3.395 | 0.0833 | 0.0000 | 1.0000 |
| 3.520 | 0.0833 | 0.0122 | 0.9878 |
| 3.715 | 0.0833 | 0.0244 | 0.9756 |
| 3.945 | 0.2500 | 0.0244 | 0.9756 |
| 4.085 | 0.2500 | 0.0366 | 0.9634 |
| 4.155 | 0.2500 | 0.0488 | 0.9512 |
| 4.275 | 0.2500 | 0.0610 | 0.9390 |
| 4.360 | 0.2500 | 0.0732 | 0.9268 |
| 4.505 | 0.2500 | 0.0976 | 0.9024 |
| 4.770 | 0.2500 | 0.1098 | 0.8902 |
| 4.935 | 0.2500 | 0.1220 | 0.8780 |
| 5.000 | 0.2500 | 0.1341 | 0.8659 |
| 5.075 | 0.2500 | 0.1463 | 0.8537 |
| 5.190 | 0.2500 | 0.1585 | 0.8415 |
| 5.320 | 0.2500 | 0.1707 | 0.8293 |
| 5.435 | 0.2500 | 0.1829 | 0.8171 |
| 5.515 | 0.2500 | 0.1951 | 0.8049 |
| 5.560 | 0.3333 | 0.1951 | 0.8049 |
| 5.630 | 0.3333 | 0.2073 | 0.7927 |
| 5.745 | 0.3333 | 0.2317 | 0.7683 |
| 5.860 | 0.3333 | 0.2439 | 0.7561 |
| 5.930 | 0.3333 | 0.2561 | 0.7439 |
| 5.995 | 0.3333 | 0.2683 | 0.7317 |
| 6.060 | 0.3333 | 0.2805 | 0.7195 |
| 6.115 | 0.3333 | 0.2927 | 0.7073 |
| 6.190 | 0.3333 | 0.3049 | 0.6951 |
| 6.255 | 0.3333 | 0.3171 | 0.6829 |
| 6.335 | 0.3333 | 0.3293 | 0.6707 |
| 6.440 | 0.3333 | 0.3415 | 0.6585 |
| 6.500 | 0.4167 | 0.3415 | 0.6585 |
| 6.540 | 0.5000 | 0.3537 | 0.6463 |
| 6.610 | 0.5833 | 0.3537 | 0.6463 |
| 6.680 | 0.6667 | 0.3537 | 0.6463 |
| 6.745 | 0.7500 | 0.3659 | 0.6341 |
| 6.805 | 0.7500 | 0.3780 | 0.6220 |
| 6.815 | 0.8333 | 0.4024 | 0.5976 |
| 6.850 | 0.8333 | 0.4146 | 0.5854 |
| 6.905 | 0.8333 | 0.4268 | 0.5732 |
| 7.000 | 0.8333 | 0.4390 | 0.5610 |
| 7.085 | 0.8333 | 0.4512 | 0.5488 |
| 7.180 * | 0.9167 | 0.4634 | 0.5366 |
| 7.440 | 0.9167 | 0.4756 | 0.5244 |
| 7.630 | 0.9167 | 0.4878 | 0.5122 |
| 7.660 | 0.9167 | 0.5000 | 0.5000 |
| 7.715 | 0.9167 | 0.5122 | 0.4878 |
| 7.795 | 0.9167 | 0.5244 | 0.4756 |
| 7.875 | 0.9167 | 0.5366 | 0.4634 |
| 7.915 | 0.9167 | 0.5488 | 0.4512 |
| 7.950 | 1.0000 | 0.5610 | 0.4390 |
| 8.015 | 1.0000 | 0.5732 | 0.4268 |
| 8.100 | 1.0000 | 0.5854 | 0.4146 |
| 8.160 | 1.0000 | 0.5976 | 0.4024 |
| 8.250 | 1.0000 | 0.6098 | 0.3902 |
| 8.365 | 1.0000 | 0.6220 | 0.3780 |
| 8.415 | 1.0000 | 0.6341 | 0.3659 |
| 8.470 | 1.0000 | 0.6463 | 0.3537 |
| 8.520 | 1.0000 | 0.6585 | 0.3415 |
| 8.615 | 1.0000 | 0.6707 | 0.3293 |
| 8.705 | 1.0000 | 0.6829 | 0.3171 |
| 8.715 | 1.0000 | 0.6951 | 0.3049 |
| 8.725 | 1.0000 | 0.7073 | 0.2927 |
| 8.785 | 1.0000 | 0.7195 | 0.2805 |
| 8.980 | 1.0000 | 0.7317 | 0.2683 |
| 9.130 | 1.0000 | 0.7439 | 0.2561 |
| 9.190 | 1.0000 | 0.7561 | 0.2439 |
| 9.285 | 1.0000 | 0.7683 | 0.2317 |
| 9.565 | 1.0000 | 0.8049 | 0.1951 |
| 9.900 | 1.0000 | 0.8293 | 0.1707 |
| 10.075 | 1.0000 | 0.8415 | 0.1585 |
| 10.210 | 1.0000 | 0.8659 | 0.1341 |
| 10.320 | 1.0000 | 0.8780 | 0.1220 |
| 10.395 | 1.0000 | 0.8902 | 0.1098 |
| 10.450 | 1.0000 | 0.9024 | 0.0976 |
| 10.535 | 1.0000 | 0.9146 | 0.0854 |
| 10.795 | 1.0000 | 0.9268 | 0.0732 |
| 11.105 | 1.0000 | 0.9390 | 0.0610 |
| 11.310 | 1.0000 | 0.9512 | 0.0488 |
| 11.540 | 1.0000 | 0.9634 | 0.0366 |
| 12.185 | 1.0000 | 0.9756 | 0.0244 |
| 15.135 | 1.0000 | 0.9878 | 0.0122 |
| 18.570 | 1.0000 | 1.0000 | 0.0000 |
|  |  |  |  |
| **18 hours** |  |  |  |
| 1.820 | 0.0000 | 0.0000 | 1.0000 |
| 2.965 | 0.0000 | 0.0122 | 0.9878 |
| 3.285 | 0.1250 | 0.0122 | 0.9878 |
| 3.885 | 0.1250 | 0.0244 | 0.9756 |
| 4.320 | 0.1250 | 0.0366 | 0.9634 |
| 4.355 | 0.1250 | 0.0488 | 0.9512 |
| 4.525 | 0.1250 | 0.0610 | 0.9390 |
| 4.735 | 0.1250 | 0.0732 | 0.9268 |
| 4.830 | 0.1250 | 0.0854 | 0.9146 |
| 4.940 | 0.1250 | 0.0976 | 0.9024 |
| 5.090 | 0.1250 | 0.1098 | 0.8902 |
| 5.345 | 0.1250 | 0.1220 | 0.8780 |
| 5.535 | 0.1250 | 0.1341 | 0.8659 |
| 5.550 | 0.2500 | 0.1341 | 0.8659 |
| 5.565 | 0.2500 | 0.1463 | 0.8537 |
| 5.610 | 0.3750 | 0.1463 | 0.8537 |
| 5.675 | 0.3750 | 0.1585 | 0.8415 |
| 5.705 | 0.3750 | 0.1707 | 0.8293 |
| 5.830 | 0.3750 | 0.2073 | 0.7927 |
| 6.040 | 0.3750 | 0.2195 | 0.7805 |
| 6.180 | 0.3750 | 0.2317 | 0.7683 |
| 6.255 | 0.3750 | 0.2439 | 0.7561 |
| 6.285 | 0.3750 | 0.2561 | 0.7439 |
| 6.305 | 0.3750 | 0.2683 | 0.7317 |
| 6.360 | 0.3750 | 0.2805 | 0.7195 |
| 6.435 | 0.3750 | 0.3049 | 0.6951 |
| 6.500 | 0.3750 | 0.3171 | 0.6829 |
| 6.620 | 0.3750 | 0.3293 | 0.6707 |
| 6.720 | 0.3750 | 0.3415 | 0.6585 |
| 6.760 | 0.3750 | 0.3537 | 0.6463 |
| 6.800 | 0.3750 | 0.3659 | 0.6341 |
| 6.845 | 0.5000 | 0.3659 | 0.6341 |
| 7.035 | 0.5000 | 0.3780 | 0.6220 |
| 7.205 | 0.5000 | 0.3902 | 0.6098 |
| 7.235 | 0.5000 | 0.4024 | 0.5976 |
| 7.255 | 0.5000 | 0.4146 | 0.5854 |
| 7.320 | 0.5000 | 0.4268 | 0.5732 |
| 7.455 | 0.5000 | 0.4390 | 0.5610 |
| 7.570 | 0.5000 | 0.4512 | 0.5488 |
| 7.615 | 0.6250 | 0.4512 | 0.5488 |
| 7.650 | 0.6250 | 0.4634 | 0.5366 |
| 7.690 | 0.6250 | 0.4756 | 0.5244 |
| 7.735 | 0.6250 | 0.5000 | 0.5000 |
| 7.815 | 0.6250 | 0.5122 | 0.4878 |
| 7.930 | 0.6250 | 0.5244 | 0.4756 |
| 8.040 | 0.6250 | 0.5366 | 0.4634 |
| 8.105 | 0.7500 | 0.5366 | 0.4634 |
| 8.230 | 0.7500 | 0.5488 | 0.4512 |
| 8.340 | 0.7500 | 0.5610 | 0.4390 |
| 8.390 * | 0.8750 | 0.5732 | 0.4268 |
| 8.435 | 0.8750 | 0.5854 | 0.4146 |
| 8.530 | 0.8750 | 0.5976 | 0.4024 |
| 8.695 | 0.8750 | 0.6098 | 0.3902 |
| 8.805 | 0.8750 | 0.6220 | 0.3780 |
| 8.860 | 0.8750 | 0.6341 | 0.3659 |
| 8.930 | 0.8750 | 0.6463 | 0.3537 |
| 9.000 | 0.8750 | 0.6585 | 0.3415 |
| 9.045 | 0.8750 | 0.6707 | 0.3293 |
| 9.155 | 0.8750 | 0.6829 | 0.3171 |
| 9.260 | 0.8750 | 0.7195 | 0.2805 |
| 9.355 | 0.8750 | 0.7317 | 0.2683 |
| 9.495 | 0.8750 | 0.7439 | 0.2561 |
| 9.630 | 1.0000 | 0.7561 | 0.2439 |
| 9.820 | 1.0000 | 0.7683 | 0.2317 |
| 9.975 | 1.0000 | 0.7805 | 0.2195 |
| 10.025 | 1.0000 | 0.7927 | 0.2073 |
| 10.100 | 1.0000 | 0.8049 | 0.1951 |
| 10.290 | 1.0000 | 0.8171 | 0.1829 |
| 10.450 | 1.0000 | 0.8293 | 0.1707 |
| 10.490 | 1.0000 | 0.8415 | 0.1585 |
| 10.655 | 1.0000 | 0.8537 | 0.1463 |
| 10.845 | 1.0000 | 0.8659 | 0.1341 |
| 10.975 | 1.0000 | 0.8780 | 0.1220 |
| 11.190 | 1.0000 | 0.8902 | 0.1098 |
| 11.370 | 1.0000 | 0.9024 | 0.0976 |
| 11.515 | 1.0000 | 0.9146 | 0.0854 |
| 11.990 | 1.0000 | 0.9268 | 0.0732 |
| 12.845 | 1.0000 | 0.9390 | 0.0610 |
| 13.380 | 1.0000 | 0.9512 | 0.0488 |
| 13.870 | 1.0000 | 0.9634 | 0.0366 |
| 15.495 | 1.0000 | 0.9756 | 0.0244 |
| 17.045 | 1.0000 | 0.9878 | 0.0122 |
| 18.390 | 1.0000 | 1.0000 | 0.0000 |
|  |  |  |  |
| **24 hours** |  |  |  |
| 1.820 | 0.0000 | 0.0000 | 1.0000 |
| 3.095 | 0.0000 | 0.0125 | 0.9875 |
| 3.670 | 0.0000 | 0.0250 | 0.9750 |
| 3.985 | 0.1111 | 0.0250 | 0.9750 |
| 4.230 | 0.2222 | 0.0375 | 0.9625 |
| 4.540 | 0.3333 | 0.0375 | 0.9625 |
| 4.835 | 0.3333 | 0.0500 | 0.9500 |
| 5.150 | 0.3333 | 0.0625 | 0.9375 |
| 5.315 | 0.3333 | 0.0750 | 0.9250 |
| 5.410 | 0.4444 | 0.0750 | 0.9250 |
| 5.465 | 0.4444 | 0.0875 | 0.9125 |
| 5.630 | 0.4444 | 0.1000 | 0.9000 |
| 5.835 | 0.4444 | 0.1125 | 0.8875 |
| 5.945 | 0.4444 | 0.1250 | 0.8750 |
| 6.025 | 0.4444 | 0.1375 | 0.8625 |
| 6.130 | 0.4444 | 0.1500 | 0.8500 |
| 6.210 | 0.4444 | 0.1625 | 0.8375 |
| 6.240 | 0.4444 | 0.1750 | 0.8250 |
| 6.265 | 0.4444 | 0.1875 | 0.8125 |
| 6.405 | 0.4444 | 0.2125 | 0.7875 |
| 6.570 | 0.4444 | 0.2250 | 0.7750 |
| 6.630 | 0.4444 | 0.2375 | 0.7625 |
| 6.700 | 0.4444 | 0.2500 | 0.7500 |
| 6.840 | 0.4444 | 0.2750 | 0.7250 |
| 6.950 | 0.4444 | 0.2875 | 0.7125 |
| 6.965 | 0.5556 | 0.2875 | 0.7125 |
| 7.010 | 0.6667 | 0.2875 | 0.7125 |
| 7.075 | 0.6667 | 0.3000 | 0.7000 |
| 7.105 | 0.6667 | 0.3125 | 0.6875 |
| 7.120 | 0.6667 | 0.3250 | 0.6750 |
| 7.190 | 0.6667 | 0.3375 | 0.6625 |
| 7.310 | 0.6667 | 0.3500 | 0.6500 |
| 7.375 * | 0.7778 | 0.3500 | 0.6500 |
| 7.420 | 0.7778 | 0.3625 | 0.6375 |
| 7.530 | 0.7778 | 0.3750 | 0.6250 |
| 7.610 | 0.7778 | 0.4000 | 0.6000 |
| 7.635 | 0.7778 | 0.4125 | 0.5875 |
| 7.665 | 0.7778 | 0.4250 | 0.5750 |
| 7.690 | 0.7778 | 0.4375 | 0.5625 |
| 7.740 | 0.7778 | 0.4500 | 0.5500 |
| 7.930 | 0.7778 | 0.4625 | 0.5375 |
| 8.095 | 0.7778 | 0.4875 | 0.5125 |
| 8.140 | 0.7778 | 0.5000 | 0.5000 |
| 8.250 | 0.7778 | 0.5250 | 0.4750 |
| 8.380 | 0.7778 | 0.5375 | 0.4625 |
| 8.475 | 0.7778 | 0.5500 | 0.4500 |
| 8.545 | 0.7778 | 0.5625 | 0.4375 |
| 8.630 | 0.7778 | 0.5750 | 0.4250 |
| 8.695 | 0.7778 | 0.5875 | 0.4125 |
| 8.805 | 0.7778 | 0.6000 | 0.4000 |
| 8.955 | 0.7778 | 0.6125 | 0.3875 |
| 9.010 | 0.7778 | 0.6250 | 0.3750 |
| 9.035 | 0.8889 | 0.6250 | 0.3750 |
| 9.060 | 0.8889 | 0.6375 | 0.3625 |
| 9.080 | 0.8889 | 0.6500 | 0.3500 |
| 9.115 | 0.8889 | 0.6625 | 0.3375 |
| 9.155 | 0.8889 | 0.6750 | 0.3250 |
| 9.205 | 0.8889 | 0.6875 | 0.3125 |
| 9.250 | 0.8889 | 0.7000 | 0.3000 |
| 9.340 | 0.8889 | 0.7125 | 0.2875 |
| 9.425 | 0.8889 | 0.7250 | 0.2750 |
| 9.515 | 0.8889 | 0.7500 | 0.2500 |
| 9.660 | 0.8889 | 0.7625 | 0.2375 |
| 9.830 | 0.8889 | 0.7750 | 0.2250 |
| 9.995 | 0.8889 | 0.7875 | 0.2125 |
| 10.130 | 0.8889 | 0.8000 | 0.2000 |
| 10.245 | 0.8889 | 0.8125 | 0.1875 |
| 10.300 | 0.8889 | 0.8250 | 0.1750 |
| 10.325 | 0.8889 | 0.8375 | 0.1625 |
| 10.430 | 0.8889 | 0.8500 | 0.1500 |
| 10.710 | 0.8889 | 0.8625 | 0.1375 |
| 11.040 | 1.0000 | 0.8625 | 0.1375 |
| 11.230 | 1.0000 | 0.8750 | 0.1250 |
| 11.290 | 1.0000 | 0.8875 | 0.1125 |
| 11.670 | 1.0000 | 0.9000 | 0.1000 |
| 12.300 | 1.0000 | 0.9250 | 0.0750 |
| 12.815 | 1.0000 | 0.9375 | 0.0625 |
| 13.195 | 1.0000 | 0.9500 | 0.0500 |
| 13.590 | 1.0000 | 0.9625 | 0.0375 |
| 14.685 | 1.0000 | 0.9750 | 0.0250 |
| 16.095 | 1.0000 | 0.9875 | 0.0125 |
| 17.670 | 1.0000 | 1.0000 | 0.0000 |
| *: optimal cutoff point | | | |

| Table II - ROC curves for the S/F ratio in relation to therapy failure in infant patients with bronchiolitis using high-flow nasal cannula | | | |
| --- | --- | --- | --- |
| S/F ratio | Sensitivity | 1 - Specificity | Specificity |
| **2 hours** |  |  |  |
| 154.000 | 0.0000 | 0.0000 | 1.0000 |
| 160.000 | 0.0000 | 0.0119 | 0.9881 |
| 168.865 | 0.0588 | 0.0119 | 0.9881 |
| 180.365 | 0.1176 | 0.0119 | 0.9881 |
| 196.220 | 0.1176 | 0.0238 | 0.9762 |
| 218.470 | 0.1176 | 0.0476 | 0.9524 |
| 236.250 | 0.1176 | 0.0595 | 0.9405 |
| 241.250 | 0.1176 | 0.0952 | 0.9048 |
| 243.750 | 0.1176 | 0.1071 | 0.8929 |
| 246.250 | 0.1176 | 0.1548 | 0.8452 |
| 248.750 | 0.1176 | 0.1786 | 0.8214 |
| 254.165 | 0.1765 | 0.1905 | 0.8095 |
| 261.595 | 0.1765 | 0.2024 | 0.7976 |
| 266.715 | 0.1765 | 0.2143 | 0.7857 |
| 270.000 | 0.2353 | 0.2262 | 0.7738 |
| 272.860 | 0.2941 | 0.2262 | 0.7738 |
| 275.715 | 0.3529 | 0.2381 | 0.7619 |
| 278.570 | 0.3529 | 0.2857 | 0.7143 |
| 281.430 | 0.4118 | 0.3095 | 0.6905 |
| 284.285 | 0.4118 | 0.3214 | 0.6786 |
| 292.855 | 0.4118 | 0.3333 | 0.6667 |
| 305.000 | 0.4118 | 0.3452 | 0.6548 |
| 311.665 | 0.4118 | 0.3571 | 0.6429 |
| 315.000 | 0.4118 | 0.3810 | 0.6190 |
| 318.335 | 0.4118 | 0.3929 | 0.6071 |
| 321.665 | 0.4706 | 0.4167 | 0.5833 |
| 325.000 * | 0.6471 | 0.5000 | 0.5000 |
| 327.335 | 0.6471 | 0.5595 | 0.4405 |
| 329.000 | 0.7059 | 0.5595 | 0.4405 |
| 331.665 | 0.7059 | 0.5952 | 0.4048 |
| 334.520 | 0.7059 | 0.6190 | 0.3810 |
| 341.070 | 0.7059 | 0.6310 | 0.3690 |
| 350.995 | 0.7059 | 0.6548 | 0.3452 |
| 365.780 | 0.7647 | 0.6548 | 0.3452 |
| 378.000 | 0.7647 | 0.7024 | 0.2976 |
| 382.000 | 0.7647 | 0.7262 | 0.2738 |
| 386.000 | 0.7647 | 0.7500 | 0.2500 |
| 390.000 | 0.8235 | 0.8095 | 0.1905 |
| 394.000 | 0.8824 | 0.8214 | 0.1786 |
| 398.000 | 0.8824 | 0.8571 | 0.1429 |
| 406.520 | 0.9412 | 0.8690 | 0.1310 |
| 432.710 | 0.9412 | 0.8810 | 0.1190 |
| 453.465 | 0.9412 | 0.8929 | 0.1071 |
| 455.845 | 0.9412 | 0.9048 | 0.0952 |
| 459.520 | 1.0000 | 0.9048 | 0.0952 |
| 464.285 | 1.0000 | 0.9524 | 0.0476 |
| 469.050 | 1.0000 | 0.9643 | 0.0357 |
| 473.810 | 1.0000 | 0.9881 | 0.0119 |
| 477.190 | 1.0000 | 1.0000 | 0.0000 |
|  |  |  |  |
| **6 hours** |  |  |  |
| 154.000 | 0.0000 | 0.0000 | 1.0000 |
| 162.955 | 0.0000 | 0.0119 | 0.9881 |
| 176.455 | 0.0625 | 0.0119 | 0.9881 |
| 190.000 | 0.1250 | 0.0119 | 0.9881 |
| 199.000 | 0.1250 | 0.0238 | 0.9762 |
| 203.335 | 0.1250 | 0.0357 | 0.9643 |
| 210.000 | 0.1250 | 0.0476 | 0.9524 |
| 214.445 | 0.1875 | 0.0476 | 0.9524 |
| 216.670 | 0.1875 | 0.0595 | 0.9405 |
| 220.000 | 0.1875 | 0.0714 | 0.9286 |
| 231.110 | 0.2500 | 0.0714 | 0.9286 |
| 241.250 | 0.2500 | 0.0952 | 0.9048 |
| 243.750 | 0.2500 | 0.1429 | 0.8571 |
| 247.500 | 0.2500 | 0.1786 | 0.8214 |
| 256.430 | 0.2500 | 0.1905 | 0.8095 |
| 263.860 | 0.2500 | 0.2024 | 0.7976 |
| 265.285 | 0.2500 | 0.2143 | 0.7857 |
| 268.570 | 0.3125 | 0.2262 | 0.7738 |
| 272.860 | 0.3125 | 0.2500 | 0.7500 |
| 277.145 | 0.3750 | 0.2619 | 0.7381 |
| 281.430 | 0.3750 | 0.2976 | 0.7024 |
| 299.495 | 0.3750 | 0.3214 | 0.6786 |
| 316.400 | 0.3750 | 0.3333 | 0.6667 |
| 318.335 | 0.3750 | 0.3571 | 0.6429 |
| 321.665 | 0.3750 | 0.4048 | 0.5952 |
| 325.000 | 0.5625 | 0.4405 | 0.5595 |
| 328.335 | 0.6250 | 0.4762 | 0.5238 |
| 332.855 | 0.6875 | 0.5238 | 0.4762 |
| 337.500 | 0.6875 | 0.5357 | 0.4643 |
| 340.335 | 0.6875 | 0.5476 | 0.4524 |
| 342.120 * | 0.7500 | 0.5476 | 0.4524 |
| 346.430 | 0.7500 | 0.5595 | 0.4405 |
| 350.925 | 0.7500 | 0.5833 | 0.4167 |
| 355.555 | 0.7500 | 0.5952 | 0.4048 |
| 361.110 | 0.7500 | 0.6071 | 0.3929 |
| 365.480 | 0.7500 | 0.6190 | 0.3810 |
| 368.615 | 0.7500 | 0.6310 | 0.3690 |
| 372.615 | 0.7500 | 0.6429 | 0.3571 |
| 378.000 | 0.7500 | 0.6548 | 0.3452 |
| 382.000 | 0.7500 | 0.7024 | 0.2976 |
| 386.000 | 0.8750 | 0.7500 | 0.2500 |
| 390.000 | 0.8750 | 0.7857 | 0.2143 |
| 394.000 | 0.8750 | 0.8333 | 0.1667 |
| 398.000 | 0.8750 | 0.8571 | 0.1429 |
| 404.165 | 0.8750 | 0.8690 | 0.1310 |
| 427.975 | 0.9375 | 0.8690 | 0.1310 |
| 452.380 | 0.9375 | 0.8810 | 0.1190 |
| 459.520 | 1.0000 | 0.9167 | 0.0833 |
| 464.285 | 1.0000 | 0.9405 | 0.0595 |
| 469.050 | 1.0000 | 0.9762 | 0.0238 |
| 473.810 | 1.0000 | 0.9881 | 0.0119 |
| 477.190 | 1.0000 | 1.0000 | 0.0000 |
|  |  |  |  |
| **12 hours** |  |  |  |
| 187.000 | 0.0000 | 0.0000 | 1.0000 |
| 200.665 | 0.0000 | 0.0122 | 0.9878 |
| 222.915 | 0.0833 | 0.0122 | 0.9878 |
| 237.500 | 0.1667 | 0.0122 | 0.9878 |
| 243.750 | 0.1667 | 0.0366 | 0.9634 |
| 247.500 | 0.1667 | 0.0732 | 0.9268 |
| 259.285 | 0.1667 | 0.0976 | 0.9024 |
| 271.430 | 0.1667 | 0.1098 | 0.8902 |
| 275.715 | 0.3333 | 0.1220 | 0.8780 |
| 278.570 | 0.3333 | 0.1707 | 0.8293 |
| 281.430 | 0.3333 | 0.1829 | 0.8171 |
| 284.285 | 0.3333 | 0.2439 | 0.7561 |
| 289.825 | 0.3333 | 0.2561 | 0.7439 |
| 300.095 | 0.3333 | 0.2683 | 0.7317 |
| 308.125 | 0.3333 | 0.2805 | 0.7195 |
| 311.665 | 0.3333 | 0.2927 | 0.7073 |
| 315.000 | 0.3333 | 0.3049 | 0.6951 |
| 318.335 | 0.3333 | 0.3415 | 0.6585 |
| 321.665 | 0.5000 | 0.3537 | 0.6463 |
| 325.000 | 0.5000 | 0.3902 | 0.6098 |
| 328.335 | 0.6667 | 0.4390 | 0.5610 |
| 331.665 | 0.6667 | 0.4512 | 0.5488 |
| 338.095 * | 0.7500 | 0.4512 | 0.5488 |
| 344.645 | 0.7500 | 0.4756 | 0.5244 |
| 348.215 | 0.7500 | 0.4878 | 0.5122 |
| 352.780 | 0.7500 | 0.5122 | 0.4878 |
| 357.410 | 0.7500 | 0.5244 | 0.4756 |
| 362.965 | 0.7500 | 0.5366 | 0.4634 |
| 367.950 | 0.7500 | 0.5610 | 0.4390 |
| 374.615 | 0.7500 | 0.5732 | 0.4268 |
| 382.000 | 0.7500 | 0.5976 | 0.4024 |
| 386.000 | 0.7500 | 0.6585 | 0.3415 |
| 390.000 | 0.9167 | 0.7073 | 0.2927 |
| 393.915 | 0.9167 | 0.7927 | 0.2073 |
| 395.915 | 0.9167 | 0.8049 | 0.1951 |
| 398.000 | 0.9167 | 0.8171 | 0.1829 |
| 406.250 | 0.9167 | 0.8537 | 0.1463 |
| 417.120 | 1.0000 | 0.8537 | 0.1463 |
| 423.915 | 1.0000 | 0.8659 | 0.1341 |
| 434.475 | 1.0000 | 0.8780 | 0.1220 |
| 450.000 | 1.0000 | 0.8902 | 0.1098 |
| 459.520 | 1.0000 | 0.9024 | 0.0976 |
| 464.285 | 1.0000 | 0.9390 | 0.0610 |
| 469.050 | 1.0000 | 0.9878 | 0.0122 |
| 472.430 | 1.0000 | 1.0000 | 0.0000 |
|  |  |  |  |
| **18 hours** |  |  |  |
| 210.110 | 0.0000 | 0.0000 | 1.0000 |
| 220.555 | 0.0000 | 0.0122 | 0.9878 |
| 232.500 | 0.1250 | 0.0122 | 0.9878 |
| 238.750 | 0.1250 | 0.0244 | 0.9756 |
| 243.750 | 0.1250 | 0.0366 | 0.9634 |
| 256.785 | 0.1250 | 0.0488 | 0.9512 |
| 270.000 | 0.1250 | 0.0732 | 0.9268 |
| 272.860 | 0.1250 | 0.0976 | 0.9024 |
| 275.380 | 0.1250 | 0.1098 | 0.8902 |
| 276.805 | 0.1250 | 0.1220 | 0.8780 |
| 278.570 | 0.2500 | 0.1585 | 0.8415 |
| 281.430 | 0.2500 | 0.1707 | 0.8293 |
| 284.285 | 0.2500 | 0.1829 | 0.8171 |
| 288.310 | 0.2500 | 0.1951 | 0.8049 |
| 302.120 | 0.2500 | 0.2073 | 0.7927 |
| 316.665 | 0.2500 | 0.2195 | 0.7805 |
| 321.665 | 0.2500 | 0.2561 | 0.7439 |
| 325.000 | 0.5000 | 0.3049 | 0.6951 |
| 328.335 | 0.5000 | 0.3171 | 0.6829 |
| 331.665 | 0.5000 | 0.3659 | 0.6341 |
| 333.905 | 0.5000 | 0.3902 | 0.6098 |
| 336.885 * | 0.6250 | 0.3902 | 0.6098 |
| 341.075 | 0.6250 | 0.4268 | 0.5732 |
| 344.645 | 0.6250 | 0.4390 | 0.5610 |
| 349.140 | 0.6250 | 0.4512 | 0.5488 |
| 354.495 | 0.6250 | 0.4634 | 0.5366 |
| 361.260 | 0.6250 | 0.4756 | 0.5244 |
| 366.025 | 0.6250 | 0.5000 | 0.5000 |
| 369.875 | 0.6250 | 0.5122 | 0.4878 |
| 376.540 | 0.6250 | 0.5244 | 0.4756 |
| 382.000 | 0.6250 | 0.5610 | 0.4390 |
| 386.000 | 0.7500 | 0.5976 | 0.4024 |
| 389.835 | 0.7500 | 0.6829 | 0.3171 |
| 391.835 | 0.7500 | 0.6951 | 0.3049 |
| 393.915 | 0.7500 | 0.7195 | 0.2805 |
| 395.915 | 0.8750 | 0.7195 | 0.2805 |
| 398.000 | 0.8750 | 0.7561 | 0.2439 |
| 404.165 | 0.8750 | 0.7927 | 0.2073 |
| 412.860 | 1.0000 | 0.7927 | 0.2073 |
| 423.910 | 1.0000 | 0.8171 | 0.1829 |
| 437.940 | 1.0000 | 0.8293 | 0.1707 |
| 448.915 | 1.0000 | 0.8415 | 0.1585 |
| 454.760 | 1.0000 | 0.8780 | 0.1220 |
| 459.520 | 1.0000 | 0.9268 | 0.0732 |
| 466.665 | 1.0000 | 0.9756 | 0.0244 |
| 472.430 | 1.0000 | 1.0000 | 0.0000 |
|  |  |  |  |
| **24 hours** |  |  |  |
| 185.000 | 0.0000 | 0.0000 | 1.0000 |
| 208.000 | 0.0000 | 0.0125 | 0.9875 |
| 235.000 | 0.1111 | 0.0125 | 0.9875 |
| 241.250 | 0.2222 | 0.0375 | 0.9625 |
| 256.965 | 0.2222 | 0.0500 | 0.9500 |
| 272.860 | 0.2222 | 0.0625 | 0.9375 |
| 275.715 | 0.3333 | 0.0750 | 0.9250 |
| 278.570 | 0.3333 | 0.0875 | 0.9125 |
| 281.430 | 0.4444 | 0.0875 | 0.9125 |
| 284.075 | 0.4444 | 0.1000 | 0.9000 |
| 289.615 | 0.5556 | 0.1000 | 0.9000 |
| 295.455 | 0.5556 | 0.1125 | 0.8875 |
| 303.485 | 0.5556 | 0.1250 | 0.8750 |
| 311.665 | 0.5556 | 0.1625 | 0.8375 |
| 315.000 | 0.5556 | 0.1750 | 0.8250 |
| 318.335 | 0.5556 | 0.1875 | 0.8125 |
| 321.665 * | 0.6667 | 0.2000 | 0.8000 |
| 325.000 | 0.6667 | 0.2625 | 0.7375 |
| 328.335 | 0.7778 | 0.3375 | 0.6625 |
| 331.070 | 0.7778 | 0.3500 | 0.6500 |
| 332.735 | 0.7778 | 0.3625 | 0.6375 |
| 336.310 | 0.7778 | 0.3875 | 0.6125 |
| 342.860 | 0.7778 | 0.4000 | 0.6000 |
| 348.215 | 0.7778 | 0.4125 | 0.5875 |
| 352.780 | 0.7778 | 0.4250 | 0.5750 |
| 356.350 | 0.7778 | 0.4375 | 0.5625 |
| 358.200 | 0.7778 | 0.4500 | 0.5500 |
| 362.965 | 0.7778 | 0.4625 | 0.5375 |
| 369.875 | 0.7778 | 0.4750 | 0.5250 |
| 374.540 | 0.7778 | 0.4875 | 0.5125 |
| 376.460 | 0.7778 | 0.5000 | 0.5000 |
| 378.460 | 0.7778 | 0.5125 | 0.4875 |
| 382.000 | 0.7778 | 0.5250 | 0.4750 |
| 386.000 | 0.7778 | 0.5625 | 0.4375 |
| 389.835 | 0.8889 | 0.6250 | 0.3750 |
| 391.835 | 0.8889 | 0.6375 | 0.3625 |
| 394.000 | 0.8889 | 0.6875 | 0.3125 |
| 398.000 | 0.8889 | 0.7250 | 0.2750 |
| 402.085 | 0.8889 | 0.7625 | 0.2375 |
| 406.250 | 1.0000 | 0.7625 | 0.2375 |
| 410.685 | 1.0000 | 0.7750 | 0.2250 |
| 417.390 | 1.0000 | 0.7875 | 0.2125 |
| 423.915 | 1.0000 | 0.8000 | 0.2000 |
| 439.235 | 1.0000 | 0.8125 | 0.1875 |
| 454.760 | 1.0000 | 0.8625 | 0.1375 |
| 459.520 | 1.0000 | 0.9250 | 0.0750 |
| 464.285 | 1.0000 | 0.9375 | 0.0625 |
| 469.050 | 1.0000 | 0.9750 | 0.0250 |
| 472.430 | 1.0000 | 1.0000 | 0.0000 |
| *: optimal cutoff point | | | |

Appendix 2 – ROC curves for ROX and S/F ratio (SpO2/FIO2) at the time of removal in the discrimination of patients without and with failure

| Table III - ROC curves for the ROX index at the time of removal in relation to therapy failure in infant patients with bronchiolitis using high-flow nasal cannula | | | |
| --- | --- | --- | --- |
| ROX index | Sensitivity | 1 - Specificity | Specificity |
| **Removal** |  |  |  |
| 2.070 | 0.0000 | 0.0000 | 1.0000 |
| 3.105 | 0.0625 | 0.0000 | 1.0000 |
| 3.190 | 0.1250 | 0.0000 | 1.0000 |
| 3.285 | 0.1875 | 0.0000 | 1.0000 |
| 3.350 | 0.2500 | 0.0000 | 1.0000 |
| 3.375 | 0.3125 | 0.0000 | 1.0000 |
| 3.450 | 0.3750 | 0.0000 | 1.0000 |
| 3.660 | 0.4375 | 0.0000 | 1.0000 |
| 3.920 | 0.5000 | 0.0000 | 1.0000 |
| 4.085 | 0.5625 | 0.0000 | 1.0000 |
| 4.135 | 0.5625 | 0.0120 | 0.9880 |
| 4.340 | 0.6250 | 0.0120 | 0.9880 |
| 4.830 | 0.6875 | 0.0120 | 0.9880 |
| 5.340 | 0.7500 | 0.0120 | 0.9880 |
| 5.875 * | 0.8125 | 0.0120 | 0.9880 |
| 6.205 | 0.8125 | 0.0241 | 0.9759 |
| 6.345 | 0.8125 | 0.0361 | 0.9639 |
| 6.715 | 0.8125 | 0.0482 | 0.9518 |
| 6.980 | 0.8125 | 0.0602 | 0.9398 |
| 7.070 | 0.8125 | 0.0723 | 0.9277 |
| 7.215 | 0.8125 | 0.0843 | 0.9157 |
| 7.310 | 0.8125 | 0.0964 | 0.9036 |
| 7.340 | 0.8125 | 0.1084 | 0.8916 |
| 7.395 | 0.8125 | 0.1205 | 0.8795 |
| 7.545 | 0.8125 | 0.1325 | 0.8675 |
| 7.660 | 0.8125 | 0.1446 | 0.8554 |
| 7.685 | 0.8125 | 0.1566 | 0.8434 |
| 7.770 | 0.8125 | 0.1687 | 0.8313 |
| 7.995 | 0.8125 | 0.1807 | 0.8193 |
| 8.200 | 0.8125 | 0.1928 | 0.8072 |
| 8.295 | 0.8125 | 0.2048 | 0.7952 |
| 8.430 | 0.8125 | 0.2169 | 0.7831 |
| 8.565 | 0.8125 | 0.2289 | 0.7711 |
| 8.660 | 0.8125 | 0.2410 | 0.7590 |
| 8.785 | 0.8125 | 0.2530 | 0.7470 |
| 8.875 | 0.8125 | 0.2651 | 0.7349 |
| 8.980 | 0.8125 | 0.2771 | 0.7229 |
| 9.165 | 0.8750 | 0.2771 | 0.7229 |
| 9.270 | 0.8750 | 0.2892 | 0.7108 |
| 9.305 | 0.8750 | 0.3012 | 0.6988 |
| 9.350 | 0.8750 | 0.3133 | 0.6867 |
| 9.400 | 0.8750 | 0.3253 | 0.6747 |
| 9.445 | 0.8750 | 0.3373 | 0.6627 |
| 9.630 | 0.9375 | 0.3373 | 0.6627 |
| 9.815 | 0.9375 | 0.3494 | 0.6506 |
| 9.885 | 0.9375 | 0.3614 | 0.6386 |
| 9.945 | 0.9375 | 0.3735 | 0.6265 |
| 10.030 | 0.9375 | 0.3855 | 0.6145 |
| 10.130 | 0.9375 | 0.3976 | 0.6024 |
| 10.180 | 0.9375 | 0.4096 | 0.5904 |
| 10.340 | 0.9375 | 0.4217 | 0.5783 |
| 10.475 | 0.9375 | 0.4337 | 0.5663 |
| 10.490 | 0.9375 | 0.4699 | 0.5301 |
| 10.575 | 0.9375 | 0.4819 | 0.5181 |
| 10.680 | 0.9375 | 0.4940 | 0.5060 |
| 10.735 | 0.9375 | 0.5060 | 0.4940 |
| 10.795 | 0.9375 | 0.5181 | 0.4819 |
| 10.855 | 0.9375 | 0.5301 | 0.4699 |
| 10.885 | 0.9375 | 0.5542 | 0.4458 |
| 10.905 | 0.9375 | 0.5663 | 0.4337 |
| 10.940 | 0.9375 | 0.5783 | 0.4217 |
| 10.980 | 0.9375 | 0.5904 | 0.4096 |
| 11.045 | 0.9375 | 0.6024 | 0.3976 |
| 11.155 | 0.9375 | 0.6145 | 0.3855 |
| 11.235 | 0.9375 | 0.6386 | 0.3614 |
| 11.280 | 0.9375 | 0.6506 | 0.3494 |
| 11.325 | 0.9375 | 0.6627 | 0.3373 |
| 11.475 | 0.9375 | 0.6747 | 0.3253 |
| 11.640 | 0.9375 | 0.6867 | 0.3133 |
| 11.800 | 0.9375 | 0.7349 | 0.2651 |
| 12.030 | 0.9375 | 0.7470 | 0.2530 |
| 12.145 | 0.9375 | 0.7590 | 0.2410 |
| 12.260 | 0.9375 | 0.7711 | 0.2289 |
| 12.415 | 0.9375 | 0.7831 | 0.2169 |
| 12.495 | 0.9375 | 0.7952 | 0.2048 |
| 12.585 | 1.0000 | 0.7952 | 0.2048 |
| 12.725 | 1.0000 | 0.8072 | 0.1928 |
| 12.865 | 1.0000 | 0.8193 | 0.1807 |
| 12.945 | 1.0000 | 0.8313 | 0.1687 |
| 13.010 | 1.0000 | 0.8554 | 0.1446 |
| 13.080 | 1.0000 | 0.8675 | 0.1325 |
| 13.205 | 1.0000 | 0.8795 | 0.1205 |
| 13.590 | 1.0000 | 0.8916 | 0.1084 |
| 13.935 | 1.0000 | 0.9036 | 0.0964 |
| 14.215 | 1.0000 | 0.9277 | 0.0723 |
| 14.505 | 1.0000 | 0.9518 | 0.0482 |
| 14.830 | 1.0000 | 0.9639 | 0.0361 |
| 15.145 | 1.0000 | 0.9759 | 0.0241 |
| 15.385 | 1.0000 | 0.9880 | 0.0120 |
| 16.560 | 1.0000 | 1.0000 | 0.0000 |
| *: optimal cutoff point | | | |

| Table IV - ROC curves for the S/F ratio at the time of withdrawal in relation to therapy failure in infant patients with bronchiolitis using high-flow nasal cannula | | | |
| --- | --- | --- | --- |
| S/F ratio | Sensitivity | 1 - Specificity | Specificity |
| **Removal** |  |  |  |
| 155.670 | 0.0000 | 0.0000 | 1.0000 |
| 158.335 | 0.0625 | 0.0000 | 1.0000 |
| 174.000 | 0.1250 | 0.0000 | 1.0000 |
| 195.110 | 0.1875 | 0.0000 | 1.0000 |
| 203.330 | 0.2500 | 0.0000 | 1.0000 |
| 208.885 | 0.3125 | 0.0000 | 1.0000 |
| 222.915 | 0.3750 | 0.0000 | 1.0000 |
| 236.250 | 0.4375 | 0.0000 | 1.0000 |
| 243.750 | 0.5000 | 0.0000 | 1.0000 |
| 248.750 | 0.5000 | 0.0120 | 0.9880 |
| 257.855 | 0.5625 | 0.0120 | 0.9880 |
| 267.140 | 0.5625 | 0.0241 | 0.9759 |
| 270.000 | 0.5625 | 0.0361 | 0.9639 |
| 278.570 | 0.6250 | 0.0361 | 0.9639 |
| 299.520 | 0.6250 | 0.0482 | 0.9518 |
| 316.665 | 0.6875 | 0.0482 | 0.9518 |
| 321.665 | 0.6875 | 0.0602 | 0.9398 |
| 326.665 | 0.7500 | 0.0964 | 0.9036 |
| 331.665 | 0.7500 | 0.1084 | 0.8916 |
| 339.880 * | 0.8125 | 0.1084 | 0.8916 |
| 348.215 | 0.8125 | 0.1205 | 0.8795 |
| 358.335 | 0.8125 | 0.1325 | 0.8675 |
| 369.335 | 0.8125 | 0.1566 | 0.8434 |
| 374.000 | 0.8125 | 0.1687 | 0.8313 |
| 376.460 | 0.8125 | 0.1807 | 0.8193 |
| 380.460 | 0.8125 | 0.1928 | 0.8072 |
| 386.000 | 0.8125 | 0.2289 | 0.7711 |
| 390.000 | 0.9375 | 0.2651 | 0.7349 |
| 394.000 | 0.9375 | 0.3735 | 0.6265 |
| 398.000 | 0.9375 | 0.4217 | 0.5783 |
| 402.085 | 0.9375 | 0.4337 | 0.5663 |
| 406.250 | 0.9375 | 0.4458 | 0.5542 |
| 408.515 | 0.9375 | 0.4578 | 0.5422 |
| 410.870 | 0.9375 | 0.4699 | 0.5301 |
| 415.215 | 0.9375 | 0.4819 | 0.5181 |
| 421.740 | 0.9375 | 0.5060 | 0.4940 |
| 429.710 | 0.9375 | 0.5301 | 0.4699 |
| 434.845 | 0.9375 | 0.5422 | 0.4578 |
| 443.180 | 0.9375 | 0.5542 | 0.4458 |
| 451.190 | 0.9375 | 0.5783 | 0.4217 |
| 454.760 | 0.9375 | 0.6265 | 0.3735 |
| 459.520 | 0.9375 | 0.6867 | 0.3133 |
| 464.285 | 0.9375 | 0.7470 | 0.2530 |
| 469.050 | 0.9375 | 0.8675 | 0.1325 |
| 473.810 | 1.0000 | 0.9759 | 0.0241 |
| 477.190 | 1.0000 | 1.0000 | 0.0000 |
| *: optimal cutoff point | | | |
