# Supplementary material for: ROX index as a predictor of failure of high-flow nasal cannula in infants with bronchiolitis
Source: Sci Rep. 2024 Jan 3;14:389. doi: 10.1038/s41598-024-51214-4 (PMC10764845; doi:10.1038/s41598-024-51214-4)
Supplement: Supplementary file 2 — Supplementary Information 2. [file 41598_2024_51214_MOESM2_ESM.docx]

**Normality of ROX index measurements**

The assumptions for generalized linear mixed models are not the same as those for an analysis of variance (ANOVA) model, they refer to the residuals generated by the fitted model and not the original data.

The procedure we followed was to adjust a model, analyze the adjustment measures and carry out an analysis of the residuals, investigating normality, independence, and constant variance.

For each variable, we chose the distribution that presented the best model fit measure (Akaike information criterion (AIC) and residuals closest to a Normal distribution. For the ROX index, the model with Normal distribution presented approximately normally distributed residues.

Analysis of adjustment measures

| 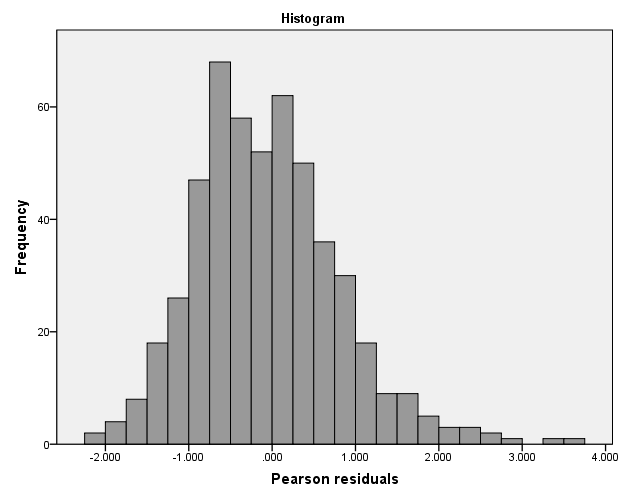 | 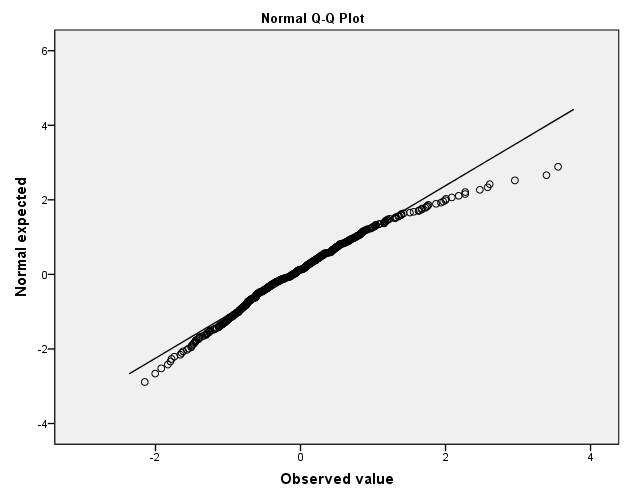 |
| --- | --- |

Supplementary figure: Analysis of adjustment measures and residuals and investigation of normality, independence, and constant variance.
